# Characterization of lactic acid bacteria derived exopolysaccharides for use as a defined neuroprotective agent against amyloid beta_1–42_-induced apoptosis in SH-SY5Y cells

**DOI:** 10.1038/s41598-020-65147-1

**Published:** 2020-05-15

**Authors:** Seda Sirin, Belma Aslim

**Affiliations:** Faculty of Science, Department of Biology, Gazi University, Ankara, Turkey

**Keywords:** Applied microbiology, Alzheimer's disease

## Abstract

Alzheimer’s disease (AD) is a disease characterized by cerebral neuronal degeneration and loss in a progressive manner. Amyloid beta (Aβ) in the brain is toxic to neurons, being a main risk factor for initiation and continuation of cognitive deterioration in AD. Neurotoxicity of Aβ origin is also linked to oxidative stress characterized by excessive lipid peroxidation, protein oxidation, changes in antioxidant systems, and cerebral DNA damage in AD. Furthermore, Aβ can induce oxidative neuronal cell death by a mitochondrial dysfunction. Cellular injury caused by oxidative stress can be possibly prevented by boosting or promoting bodily oxidative defense system by supplying antioxidants in diet or as medications. However, most synthetic antioxidants are found to have cytotoxicity, which prevents their safe use, and limits their administration. For this reason, more attention has been paid to the natural non-toxic antioxidants. One of the most promising groups of non-toxic antioxidative compounds is thought to be polysaccharides. This study investigated the characterization and protective action exerted by exopolysaccharides (EPSs) originated from *Lactobacillus delbrueckii* ssp. *bulgaricus* B3 and *Lactobacillus plantarum* GD2 to protect from apoptotic activity exerted by Aβ_1-42_ among SH-SY5Y cells. We characterized EPSs by elemental analysis, FTIR, AFM, SEM, and XRD. The antioxidant effects of EPSs were determined by the DPPH free radical scavenging activity, hydroxyl radical scavenging activity, metal ion chelating activity, lipid peroxidation inhibitory activity, and superoxide anion scavenging activity method. The protective effects of EPSs were determined by flow cytometry and RT-PCR. Mannose ratio, molecular weight, functional groups, surface morphology, and amorphous character structure of EPSs are thought to play a role in the protective effect of EPSs. EPSs reduced apoptotic activity of Aβ_1-42_ in addition to their depolarizing effect on mitochondrial membrane potential in concentration-dependent manner. These observations contribute the inclusion of EPSs among the therapeutic options used to manage various neurological disorders in the traditional medicine in a scientific manner, indicating that EPSs may be promising natural chemical constituents that need advanced research and development for pharmacological therapy of AD.

## Introduction

Alzheimer’s disease (AD) is a disease characterized by cerebral neuronal degeneration and loss in a progressive manner; these events show parallelism with two most striking neuropathological signs of AD, i.e. the formation of neurofibrillary tangles and senile plaques^1^. Amyloid beta (Aβ) fibrils are formed in multiple steps, which follow the monomeric Aβs being oligomerized and aggregated, where the peptide goes through a conformational change from an alpha-helical to a beta-pleated sheet architecture. It has been recently shown that cerebral neurotoxicity arises from the effects of soluble oligomers of Aβ^2^. Multiple studies both performed *in vitro* and *in vivo* have demonstrated that Aβ can directly be fatal for neurons. Neurotoxicity of Aβ origin is also linked to oxidative stress characterized by excessive lipid peroxidation, protein oxidation, changes in antioxidant systems, and cerebral DNA damage in AD. Furthermore, Aβ can induce oxidative neuronal cell death by mitochondrial dysfunction^3^.

Cellular injury caused by oxidative stress can be possibly prevented by boosting or promoting bodily oxidative defense system by supplying antioxidants in diet or as medications^1^. However, most synthetic antioxidants are found to have cytotoxicity, which prevents their safe use, and limits their administration. For this reason, more attention has been paid to the natural non-toxic antioxidants^4^.

One of the most promising groups of non-toxic antioxidant compounds is thought to be polysaccharides^5^. Polysaccharides widely occur in algae, animals, microorganisms (fungi and bacteria), and plants. Due to their wide availability and rheological properties, polysaccharides are widely used in textile, dairy, cosmetics, and other industries, production of drugs and pharmaceutical agents, for environmental purposes such as remediation, flocculation etc.^6,7^.

Recently, exopolysaccharides (EPSs) formed by various strains of lactic acid bacteria (LAB) have been increasingly reported to exert powerful biological actions such as effects against oxidation, immunomodulating, antitumor, antibiofilm and antibacterial properties^8^. Their thorough characterization is vital for fully studying and understanding their functions^9^.

In the literature, there are several studies carried out to determine the protective effects of LAB on AD. Nimgampalle & Kuna^10^ attempted to study the anti-Alzheimer actions of *Lactobacillus plantarum* MTCC1325 in albino rats with AD induced by D-Galactose (D-Gal). It has been recently shown that the *L. plantarum as* compared with *L. pentosus* protected against memory defect in D-Gal and scopolamine-induced AD in mice^11,12^. Furthermore, it has been shown elsewhere that older rats had their learning and memory skills improved by *L. plantarum* NDC75017^13^.

In the literature, studies related to EPSs originating from LAB are mostly associated with antioxidant, immunomodulating, antitumor, antibiofilm, and antibacterial properties^4,8,14^. No study has investigated the effects of EPSs originated from LAB in AD.

In the light of the above-mentioned points, SH-SY5Y cells were employed in this study to investigate any protective effect of EPSs produced by *Lactobacillus delbrueckii* ssp. *bulgaricus* B3 and *Lactobacillus plantarum* GD2 on Aβ_1-42_-induced cell death, mitochondrial dysfunction, as well as the activation of the intrinsic cell death pathway. The characterization of the EPSs was carried out in order to determine the structures of the EPSs and their possible effects. The results enabled a more thorough appreciation of EPSs protective effects.

## Materials and methods

### Bacterial strains, media and culture conditions

This research studied *Lactobacillus delbrueckii* ssp*. bulgaricus* B3 (isolated from yoghurt) and *Lactobacillus plantarum* GD2 (isolated from stoll) strains at Gazi University, Biotechnology Laboratory Collection for Type Cultures. The morphological and cultural characteristics, catalase test, and the API 50 CHL kit (BioMerieux) analyzed by API LAB plus software version 4.0 databases (data not shown) were used to classify strains. Additionally, strains were confirmed using analysis the sequence of 16 S ribosomal RNA (rRNA). Amplification of 16 S rRNA gene regions by polymerase chain reaction (PCR) and sequencing were done using Uni27F (20 bp) 5′ AGAGTTTGATCCTGGCTCAG 3′ and Uni1492R (19 bp) 5′ GGTTACCTTGTTACGACTT 3′. The basic local alignment search tool (BLAST) algorithm was utilized to compare the obtained sequences with those deposited in the GenBank database. Strains were cultured in de Man Rogosa Sharpe (MRS) broth (Oxoid). Strains were stored in MRS broth with 10% glycerol (Sigma-Aldrich) at −40 °C and regenerated twice prior to their use in the applications^15,16^.

### Isolation and quantification of exopolysaccharides (EPSs)

The cultures were boiled at 100 °C for 15 min. They were then cooled and treated with 17% (v/v) of 85% trichloroacetic acid (TCA) (Merck Millipore) solution and centrifuged at 13.000 rpm for 20 min (Nuve) to eliminate cells and proteins. The EPSs were subjected to precipitation with 1 volume of cold absolute ethanol (Sigma-Aldrich) and then centrifuged at 13.000 rpm for 15 min. The pellet with EPSs in it was dissolved in deionized water. EPSs were re-precipitated with ethanol, and the EPS solution in deionized water was lyophilized. The lyophilized-EPSs (l-EPSs) total carbohydrate amount was calculated with the phenol-sulfuric acid method with glucose (Merck Millipore) as standard^16–18^.

### Elemental analysis of l-EPSs

l-EPSs elemental composition was analyzed using an elemental analyzer (Thermo Scientific Flash 2000) without acid hydrolysis. Carbon (C), hydrogen (H), nitrogen (N), and oxygen (O) were analyzed in order to assess the elemental composition of l-EPSs. The analysis was carried out by Düzce University, Scientific and Technological Researches, Research and Application Center, Environmental and Chemistry Laboratory, Düzce, Turkey.

### Fourier transform infrared (FTIR) spectroscopy of l-EPSs

FTIR spectrum for l-EPSs sample was acquired using Shimadzu IRprestige 21 in transmittance mode to investigate different functional groups. Compressed discs that are 3 mm in diameter formed via admixing 2 mg l-EPSs and 200 mg potassium bromide (KBr) (Merck Millipore), with the spectrum being corrected for KBr background. Then, the pellets were scanned in the range of 4000–500 cm^−1^ with a resolution 4 cm^−1^ and utilizing 32 scans. The analysis was carried out by Düzce University, Scientific and Technological Researches Research and Application Center, Environmental and Chemistry Laboratory, Düzce, Turkey^19^.

### Atomic force microscopy (AFM) of l-EPSs

Glass slides were treated with a mixture of 15 mL of hydrochloric acid (HCl) (Merck Millipore) and 5 mL of nitric acid (HNO_3_) (Merck Millipore) for 30 min. Thereafter, they were subjected to a mixture of 20 mL of sulfuric acid (H_2_SO_4_) (Merck Millipore) and 5 mL of hydrogen peroxide (H_2_O_2_) (Sigma-Aldrich) for 30 min. Afterward, the slides were rinsed with deionized water, in which they were subsequently stored until use. l-EPSs were dissolved in deionized water to form fresh l-EPS solutions. Approximately 10 𝜇L of l-EPSs was poured on the glass slide and dried at room temperature. The AFM images were then acquired with Park Systems XE-100E in semi-contact mode with a cantilever with a constant force of 5.5–22.5 N/m. The freestanding gold (Au) and silver (Ag) films were put on a glass surface. The silicon (Si) probes coated with Au had the sizes below: chip size, 3.6∗1.6∗0.4 mm, the radius of curvature, 10 nm; tip height 10–15 𝜇m. The Nova software was used to analyze the images. The analysis was carried out by Düzce University, Scientific and Technological Researches, Research and Application Center, Material and Surface Laboratory, Düzce, Turkey^19,20^.

### Scanning electron microscopy (SEM) of l-EPSs

The SEM images of l-EPSs were captured using FEI Quanta FEG 250. The l-EPSs (5 mg) were fixed to the SEM specimen stubs with double-sided tape, and then coated with a layer of Au with a thickness of 10 nm. The samples were observed in a SEM at an accelerating voltage of 10.0 kV. The analysis was carried out by Düzce University, Scientific and Technological Researches, Research and Application Center, Material and Surface Laboratory, Düzce, Turkey.

### X-ray diffraction (XRD) analysis of l-EPSs

To determine the crystalline nature of the l-EPSs by examining the physical properties of l-EPSs with the use of slow-scan in different ranges of two-theta angles (10–90◦), an X-ray diffractometer (Rigaku Smart Lab) device capable of emitting a PW3123/00 curved Ni-filtered CuK (=1.54056 A) radiation which is generated at 40 kV and 30 mA with liquid nitrogen cooled solid-state germanium detector was used. The analysis was carried out by Bartın University, Research Laboratory, Scientific and Technological Research Application and Research Center, Bartın, Turkey.

### Antioxidant activity of l-EPSs

#### 2,2-Diphenyl-1-picrylhydrazyl (DPPH) free radical scavenging activity

Different concentrations of the l-EPSs in methanol (Merck) at a quantity of 1 mL were added to 1 mL of a 0.004% methanol solution of DPPH (Sigma-Aldrich). The mixture was then sloshed powerfully and left in an upright position for 30 min in the dark, followed by the measurement of its absorbance at 517 nm against a blank. The scavenging activity on DPPH radical was calculated using the formula: Scavenging activity (%) = (1 − A_sample_ − A_0_/A_blank_) × 100; where *A*_0_ is the sample’s absorbance under identical conditions with A_sample_ with methanol substituted for DPPH radical solution. Deionized water was utilized as the blank and ascorbic acid (0.5 mg/mL) (Sigma-Aldrich) as the positive control^21^.

### Hydroxyl radical scavenging activity

The hydroxyl radical scavenging action of l-EPSs was studied based on the method described by Leung *et al*.^22^ and Xiao *et al*.^23^ In summary, the reaction mixture, which contained 1 mL sample solution (100–1250 µg/mL), 1 mL 1,10-phenanthroline (0.75 mM) (Sigma-Aldrich), 1.5 mL 0.15 M sodium phosphate buffer (pH 7.4) (Merck), 1 mL iron (II) sulfate (FeSO_4_) (0.75 mM) (Sigma-Aldrich), and 1 mL H_2_O_2_ (0.01%, v/v), was shaken and left for incubation at a temperature of 37 °C and for 30 min. The measurement of the mixture’s absorbance was performed at 536 nm. The formula below was used to determine the scavenging activity on hydroxyl radical: Scavenging activity (%) = (1 − A_sample_ − A_0_/A_blank_) × 100; where A_sample_ and A_blank_ denote the absorbance of the sample and the control (water instead of the sample), respectively. *A*_0_ denotes water’s absorbance instead of that of H_2_O_2_ and the sample in the assay system. Ascorbic acid (0.5 mg/mL) served as positive control.

### Lipid peroxidation inhibitory activity

A mixture was formed by admixing a total of 0.4 mL of plasma, 0.1 mL of 0.5 mM FeSO_4_, 0.1 mL of 0.5 mM H_2_O_2_, and 0.2 mL of l-EPSs (100–1250 µg/mL), which was incubated at 37 °C For 12 h. After the completion of the incubation period, 375 µL of 4% tricarboxylic acid (TCA) (Merck) and 75 µL of 0.5 mM butylhydroxytoluene (BHT) (Sigma-Aldrich) was added to the reaction solution, followed by keeping the admixture in an ice bath for 5 min. A supernatant was formed after centrifugation of the admixture at 5000 rpm for 15 min. Then a total of 0.2 mL of 0.6% thiobarbituric acid (TBA) (Sigma-Aldrich) was added. The obtained mixture was left for incubation at 95 °C for 30 min and left to cool. Then, its supernatant was obtained after centrifugation at 5000 rpm for 15 min. This was followed by measuring the mixture’s absorbance at 532 nm. The inhibitory activity exerted by lipid peroxidation was calculated by the following formula: Inhibitory activity (%) = (1 − A_sample_ − A_0_/A_blank_) × 100. Ascorbic acid (0.5 mg/mL) was added as the positive control^24^.

### Metal ion chelating activity

A mixture was formed by adding a total of 1 mL of the l-EPSs (100–1250 µg/mL), 3.7 mL of methanol, and 0.1 mL of 2 mmol/L ferrous chloride (FeCl_2_) (Sigma-Aldrich). The reaction was started by adding 0.2 mL of 5 mmol/L ferrozine (Sigma-Aldrich). This was followed by shaking the mixture vigorously and putting it in an upright position at room temperature for 10 min. After the equilibrium is reached, the solution’s absorbance was determined at 562 nm. The formula below defines the chelating ability of metal ion: Scavenging activity (%) = (1 − A_sample_ − A_0_/A_blank_) × 100; where A_0_ is the sample absorbance under identical conditions as A_sample_ with water substituted for FeCl_2_ solution. Deionized water was added as the blank and ethylenediaminetetraacetic acid (EDTA)-sodium (Na) (0.5 mg/mL) as the positive control^25,26^.

### Superoxide anion scavenging activity

The superoxide anion scavenging activity was studied as described by Ye *et al*.^27^ and Xiao *et al*.^23^ 3 mL of 0.1 M sodium phosphate buffer (pH 7.4) that contained 156 μM nicotinamide adenine dinucleotide (NADH) (Merck), 52 μM nitro blue tetrazolium (NBT) (Merck) and 20 μM phosphate buffered saline (PBS) (Thermo Fisher Scientific) was used to form the superoxide radical. The addition of 1 mL sample (100–1250 µg/mL) was followed by the incubation of the mixture at 25 °C for 5 min and measurement at 560 nm. The following formula was used to determine the scavenging activity on superoxide radical: Scavenging activity (%) = (1 − A_sample_ − A_0_/A_blank_) × 100. Deionized water was added as the blank control and ascorbic acid (0.5 mg/mL) as positive control.

### Cell culture and treatment

SH-SY5Y cells were cultured in Dulbecco’s Modified Eagle Medium (DMEM) (Gibco) supplemented with 10% heat-inactivated fetal bovine serum (FBS) (Gibco), 100 U/mL of penicillin (Gibco), and 100 μg/mL of streptomycin (Gibco). The cultures were maintained in a humidified atmosphere of 5% carbon dioxide (CO_2_)/95% air at 37 °C, and the culture medium was replaced every 2–3 days. SH-SY5Y cells were seeded into 96- or 6-well plates (Corning), which were then allowed to equilibrate for 24 h before experiments. Amyloid beta (Aβ)_1–42_ (Sigma-Aldrich) was dissolved in deionized water at a concentration of 100 µM. The stock solution was diluted to the desired concentrations immediately before use. In the establishment of Aβ_1-42_ group, cells were induced by 10 μM of Aβ_1-42_ for 24 h. In protective experiments of l-EPSs, cells were pretreated with various concentrations (100, 250, 500, 1000, and 1250 μg/mL) of l-EPSs dissolved in serum-free media for 24 h respectively, and then exposed to 10 μM (final concentration) of Aβ_1-42_ in the presence of l-EPSs for another 24 h. The control cells were added with the same medium without Aβ_1-42_. All experiments were repeated three times for each treatment condition^19,20^.

### Determination of cell viability

Cell viability was determined using the thiazolyl blue tetrazolium bromide (MTT) assay. After incubation with the indicated l-EPSs, all the media in 96-well plates were removed and replaced with serum-free media containing 0.5 mg/mL of MTT (Sigma-Aldrich). The plate was further incubated with 5% CO_2_ for 4 h at 37 °C, then MTT solution was removed and cells were lysed with 200 μL dimethyl sulfoxide (DMSO) (Sigma-Aldrich). The plate was then placed on an orbital shaker (VWR) for 30 min before determining the absorbance at 570 nm using a microplate reader (Biotek Instruments). The absorbance values were expressed as a percentage of untreated control cells (control = 100%)^28^.

### Thioflavin T (ThT) assay of Aβ_1-42_ fibril and aggregate formation

ThT (Sigma-Aldrich) (10 μM in PBS) was admixed with Aβ_1–42_ (10 μM), either alone or combined with five different concentrations of each l-EPS. Plate incubation took place at 37 °C in a fluorescence microplate reader (Bio-Tek) that had a excitation at 446 nm and an emission at 490 nm.

### AFM imaging

The procedures similar to the ThT assay were used to prepare samples for AFM. Glass slides were treated with a mixture of 15 mL of HCl and 5 mL of HNO_3_ for 30 min. Thereafter, they were subjected to a mixture of 20 mL of H_2_SO_4_ and 5 mL of H_2_O_2_ for 30 min. Afterward, the slides were rinsed with deionized water, in which they were subsequently stored until use. Aβ_1-42_ and l-EPSs were dissolved in deionized water to form fresh Aβ_1-42_ and l-EPS solutions. Approximately 10 𝜇L of Aβ_1-42_ and l-EPSs was poured on the glass slide and dried at room temperature. The AFM images were then acquired with Park Systems XE-100E in semi-contact mode with a cantilever with a constant force of 5.5-22.5 N/m. The freestanding Au and Ag films were put on a glass surface. The Si probes coated with Au had the sizes below: chip size, 3.6∗1.6∗0.4 mm, the radius of curvature, 10 nm; tip height 10–15 𝜇m. The Nova software was used to analyze the images^19,20^.

### Detection of apoptotic cells using flow cytometry

The cells were collected by centrifugation 24 h after Aβ_1-42_ exposure and washed with PBS. Then the cells resuspended in 1x annexin-binding buffer (Abcam) at a concentration 1 × 10^6^ cells/mL. 5 μL of the annexin V-fluorescein isothiocyanate (FITC) (Abcam) and 5 μL of propidium iodide (PI) (Abcam) were added to each 100 µL of the cell suspension (up 1 × 10^6^ cells). The cells were incubated in the dark at room temperature for 15 min. After the incubation period, 400 μL of 1x annexin-binding buffer was added. The labeled cells were then analyzed using ACEA NovoCyte flow cytometry. In each sample, 10.000 cells were analyzed, and the percentage of live and apoptotic cells were calculated by ACEA NovoExpress software.

### Detection of mitochondrial membrane potential using flow cytometry

Tetraethylbenzimidazolylcarbo-cyanine iodide (JC-1) (100 μL) (Cayman Chemical) was added to the cells (up 1 × 10^6^ cells) after the treatment with l-EPSs plus Aβ_1-42_ as described above. At the end of the 30 min-culture at 37 °C, the cells were collected by pipetting, washed twice with PBS and then analyzed using the ACEA NovoCyte flow cytometer. In each sample, 10.000 cells were analyzed, and the percentages of intact and damaged mitochondria were calculated using the ACEA NovoExpress software.

### Reverse transcription-polymerase chain reaction (RT-PCR)

Total RNA was extracted from cells cultured in the 6-well plates using The RNeasy Mini kit (Qiagen) according to the manufacturer’s instructions. First-strand complementary DNAs (cDNA) were generated by reverse transcription from RNA samples using oligo (dT) with QuantiTect Reverse Transcription kit (Qiagen). Primer sequences were shown in Table 1. Following cDNA synthesis, PCR was carried out at 95 °C for 5 min, 95 °C for 10 sec, and 60 °C for 30 sec for 40 cycles. Electrophoresis in 2% agarose gel was done on PCR products, which were visualized by ethidium bromide (EB) (Sigma-Aldrich) staining. The relative expression was quantified densitometrically using the QuantStudio 3 Real-Time PCR (Applied Biosystems), and calculated based on the reference expression level of glyceraldehyde 3-phosphate dehydrogenase (GAPDH).

**Table 1:** Primers used in RT-PCR.

Gene name	Forward primer 5′→3′	Reverse primer 5′→3′	Product (bp)
Bax	GGAGCTGCAGAGGATGATTG	GGCCTTGAGCACCAGTTT	151
Bcl-2	GTGGATGACTGAGTACCTGAAC	GAGACAGCCAGGAGAAATCAA	125
Caspase-3	GAGCCATGGTGAAGAAGGAATA	TCAATGCCACAGTCCAGTTC	162
Caspase-7	CGAAACGGAACAGACAAAGATG	TTAAGAGGATGCAGGCGAAG	169
Caspase-8	GCCCAAACTTCACAGCATTAG	GTGGTCCATGAGTTGGTAGATT	160
Caspase-9	CGACCTGACTGCCAAGAAA	CATCCATCTGTGCCGTAGAC	153
Cyctochrome *c*	GGAGAGGATACACTGATGGAGTA	GTCTGCCCTTTCTTCCTTCTT	102
Gapdh	TGAACGGGAAGCTCACTGG	TCCACCACCCTGTTGCTGTA	307

### Statistics

All experiments were performed in triplicate, and mean values are presented. The results were given as mean ± standard deviation (SD). Statistical analyses were made using SPSS version 16.0. Pearson’s correlation analysis was used to describe the statistical significance of differences between the values.

## Results and discussion

The identification of the isolates was done via sequencing each one’s 16 S rRNA gene region, resulting in the amplification of a fragment of 1492 bp. The isolates were shown to be lactobacilli and were ≥ 99% similar to strain types deposited in the GenBank database (gene bank accession number FJ915697.1 and AB112083.1). As the results of the sequence analysis, isolates were identified as *Lactobacillus delbrueckii* ssp. *bulgaricus* B3, and *Lactobacillus plantarum* GD2.

The amounts of l-EPSs of *L. delbrueckii* ssp. *bulgaricus* B3 and *L. plantarum* GD2 produced were 435 and 385 mg/L, respectively. *L. delbrueckii* ssp. *bulgaricus* B3 produced the highest amount of l-EPS. In previous studies, the amount of EPS varies from 25 to 132 mg/L for *L. lactis* ssp. *cremoris* and *L. rhamnosus* C83, and 130 to 250 mg/L for *L. casei* CG11 and *L. delbrueckii* ssp. *bulgaricus* NCFB 2772, respectively^29–31^. A comparative analysis of our and previous studies^29–31^ showed that l-EPSs production of *Lactobacillus* spp. was comparatively higher.

Mannose (88.25%), glucose (9.54%), sucrose+maltose (1.10%), fructose (1.04%), and n-acetyl glucosamine (0.07%) were determined in *L. delbrueckii* ssp. *bulgaricus* B3 and mannose (71.03%), glucose (25.97%), arabinose (2.73%), and n-acetyl glucosamine (0.27%) in *L. plantarum* GD2 in our previous study^32^. The presence of different sugar moieties suggests that the l-EPSs were heteropolysaccharides. Mannose has been identified as the predominant monomer in l-EPSs. According to a review focusing on the functional properties of yeast-derived EPSs, EPSs that contain mannose more than 50% of their total content are regarded as biologically active^33^. In a study where the *in vitro* effect of a series of truncated derivatives of the oligosaccharide against Aβ peptide toxicity was investigated, Jiang *et al*.^34^ reported protective effects of β-(1,4)-*D*-mannans isolated from a marine plant. For l-EPS obtained from *L. delbrueckii* ssp. *bulgaricus* B3, mannose ratio is higher than that observed for l-EPS obtained from *L. plantarum* GD2. For this reason, biological activity of the l-EPS obtained from *L. delbrueckii* ssp. *bulgaricus* B3 may be higher.

The molecular weight of l-EPS from *L. delbrueckii* ssp. *bulgaricus* B3 was found to be 1.2 × 10^4^ and 3.5 × 10^2^ Da. The molecular weight of l-EPS from *L. plantarum* GD2 was found to be 2.4 × 10^3^ and 2.3 × 10^2^ Da in our previous study^32^. EPSs with low molecular weight (≤10^4^ Da) can reportedly pass readily through the membranes of host cells^35^. In contrast, bioactive EPSs with greater molecular weight have been reported to exert significant antitumor, immunomodulatory, antioxidant, and neuroprotective activities^36^. Ayyash *et al*.^37^ demonstrated a larger molecular weight (3.8 × 10^5^ Da) for EPS-C70 produced by the new probiotic *L. plantarum* C70 which was isolated from camel milk. EPS-C70 also showed significant bioactivity. Cheng *et al*.^36^ tested the protective effects of purified *Hericium erinaceus* (HE) polysaccharides (HEPS) with a structure of dual high molecular weight polysaccharides (1.7 × 10^5^ Da and 1.1 × 10^5^ Da) against Aβ-induced neurotoxicity in rat pheochromocytoma PC12 cells. For l-EPS obtained from *L. delbrueckii* ssp. *bulgaricus* B3, molecular weight is higher than that observed for l-EPS obtained from *L. plantarum* GD2. For this reason, biological activity of the l-EPS obtained from *L. delbrueckii* ssp. *bulgaricus* B3 may be higher.

l-EPSs anomeric H1 protons showed a chemical shift at δ 4.8, δ 4.9, and δ 5.2 ppm. H1 proton of α-D-mannose (5.2 ppm) and H2 proton of α-D-glucose (4.0 ppm protons) interacted with each other to produce the observed peaks. Additionally, the H3 proton of α-D-mannose and H1 proton of β-D-mannose also showed signs of interaction. According to these findings, all study lactobacilli produced l-EPSs with the same type of sugar bond, namely β-H1-H3 (β-D-Mannose-α-D-Mannose)/α-H1-H2 (α-D-Mannose-α-D-Glucose), which were named as β−1,3 (β-D-Mannose-α-D-Mannose) and α-1,2 (α-D-Mannose-α-D-Glucose), respectively. Also, α(1–2), and β(1–3) bonds were determined in the structure of l-EPSs in our previous study^32^. In a study by Tsai *et al*.^38^ polysaccharide–peptide complexes were detected in *Cordyceps militaris* (CPSPs) and showed a characteristic inhibitory action against *acetylcholinesterase* (AchE). Polysaccharide–peptide complexes were identified by polysaccharide–peptide bond categories released by β-elimination reaction. The absorption peak at 825 cm^−1^ pointed to an α-glycosidic bond while absorption at 893 cm^−1^ was an sign of a β-glycosidic bond, although the bands at 930 cm^−1^, 1038 cm^−1^, 1078 cm^−1^, and 1161 cm^−1^ also reflected β(1–3) glycosidic bonds. The bonds of α(1–2), and β(1–3) in l-EPSs from *L. delbrueckii* ssp. *bulgaricus* B3 and *L. plantarum* GD2 were compatible with the literature^38^.

The C, H, N, and O content (w/w%) of the l-EPSs were determined using elemental analysis **(**Table 2**)**. High percentages of C and O signify a composition mainly consisting of sugar moieties. In the study of Goh *et al*.^39^, the amounts of C, H, N, and O in EPS from *L. delbrueckii* subsp. *bulgaricus* NCFB 2483 obtained by elemental analysis were 39.1, 6.2, 2.8, and 50.3, respectively. In the study of Wang *et al*.^40^, the amounts of C, H, N, and S in crude L3 EPS (C-EPS) from *L. sakei* L3 obtained by elemental analysis were 39.3, 6.1, 0.4, and 0.1, respectively. Usually, polysaccharide-rich samples do not contain or show only small amounts of N^41^. In this study the amount of N in l-EPSs by elemental analysis was 5.94 and 1.58%. According to the data we obtained from our previous study^32^ (due to the presence of n-acetyl glucosamine in the monomer compositions of l-EPSs), N may be caused by n-acetyl glycosamine. Du *et al*.^41^ suggested that N originated from proteins or chitin. These proteins are probably bound to soluble glucans. Chabot *et al*.^42^ and Van Calsteren *et al*.^43^ revealed that N was not present in EPS produced by *L. rhamnosus* RW-9595M and *L. rhamnosus* strains RW-9595M and R, respectively. The amounts of C, H, N, and O in l-EPSs from *L. delbrueckii* ssp. *bulgaricus* B3 and *L. plantarum* GD2 were compatible with the literature^39,41^.

**Table 2:** Elemental analysis of l-EPSs.

Sample	%			
	N	C	H	O
l-EPS_B3_	5.94	44.81	6.56	42.69
l-EPS_GD2_	1.58	23.33	6.44	68.65

**Figure 1:**
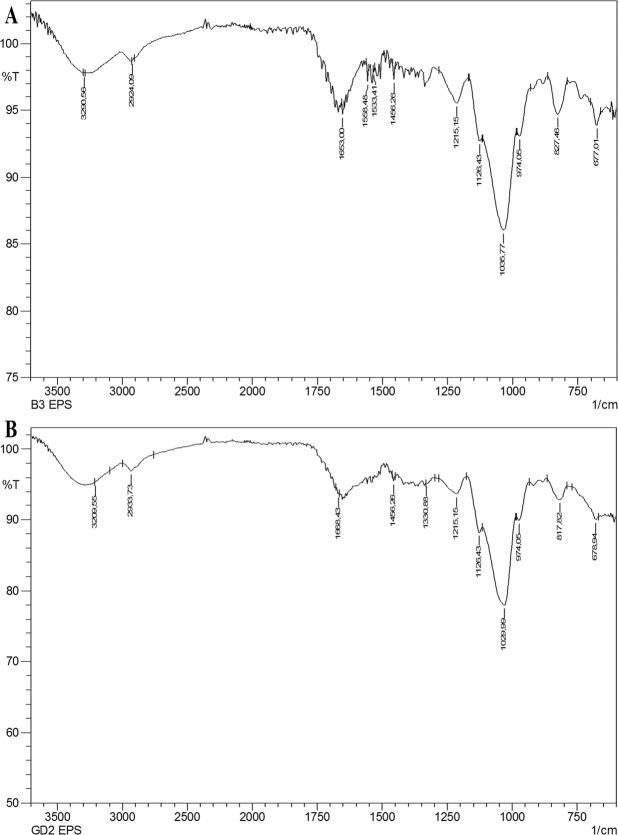
FTIR spectrum of l-EPS_S_ [l-EPS_B3_ (**A**) and l-EPS_GD2_ (**B**)].

It was shown by the l-EPS samples FTIR spectra that there was a high number of O-H groups within the structure of polysaccharides, which corresponded to hydroxyl group vibration of carbohydrates as indicated by broad stretching in the area of 3300 cm^−1^. The C-H stretching of methyl or methylene groups typically found in hexoses such as glucose or galactose, or deoxyhexoses such as rhamnose or fucose, is a phenomenon to which the absorption band at 2850–2960 cm^−1^ can be assigned. Stretching vibration of carbonyl group (C=O) was the source of the absorptions around the region of 1735 cm^−1^; the C=O stretching vibration caused the stretching bands around the region of 1650–1660 cm^−1^; and the stretching vibration of the carboxyl group (C=O) caused the absorptions around the region of 1200–1400 cm^−1^. The wave number region between 800 and 1200 cm^−1^ is denominated fingerprint region characterizing various separate polysaccharides. The absorption bands corresponding to the region of 1200 cm^−1^ indicated the existence of sugar monomers. The vibration of the C-O-C bond was held responsible for bands observed around the region of 974 cm^−1^. The band corresponding to the region of 836 cm^−1^ is characteristic for α*-*D glucan **(**Fig. 1A,B**)**. The functional group analysis with the vibration frequencies performed using the FTIR spectra analysis revealed that the two l-EPSs samples are carbohydrates when compared to the results of other EPSs reported in the literature^44^. *L. plantarum* YW11 of Tibet kefir origin and *L. helveticus* MB2-1 of say ram ropy fermented milk origin demonstrated similar FTIR peak range^45,46^. The biological activity of EPSs is shown to depend on their functional groups such as hydroxyl, amino, carbonyl, and carboxyl groups (polyanionic functional groups)^47,48^. For l-EPS obtained from *L. delbrueckii* ssp. *bulgaricus* B3, the absorption of the polyanionic functional groups due to stretching is greater than that observed for l-EPS obtained from *L. plantarum* GD2. For this reason, biological activity of the l-EPS obtained from *L. delbrueckii* ssp. *bulgaricus* B3 may be higher.

**Figure 2:**
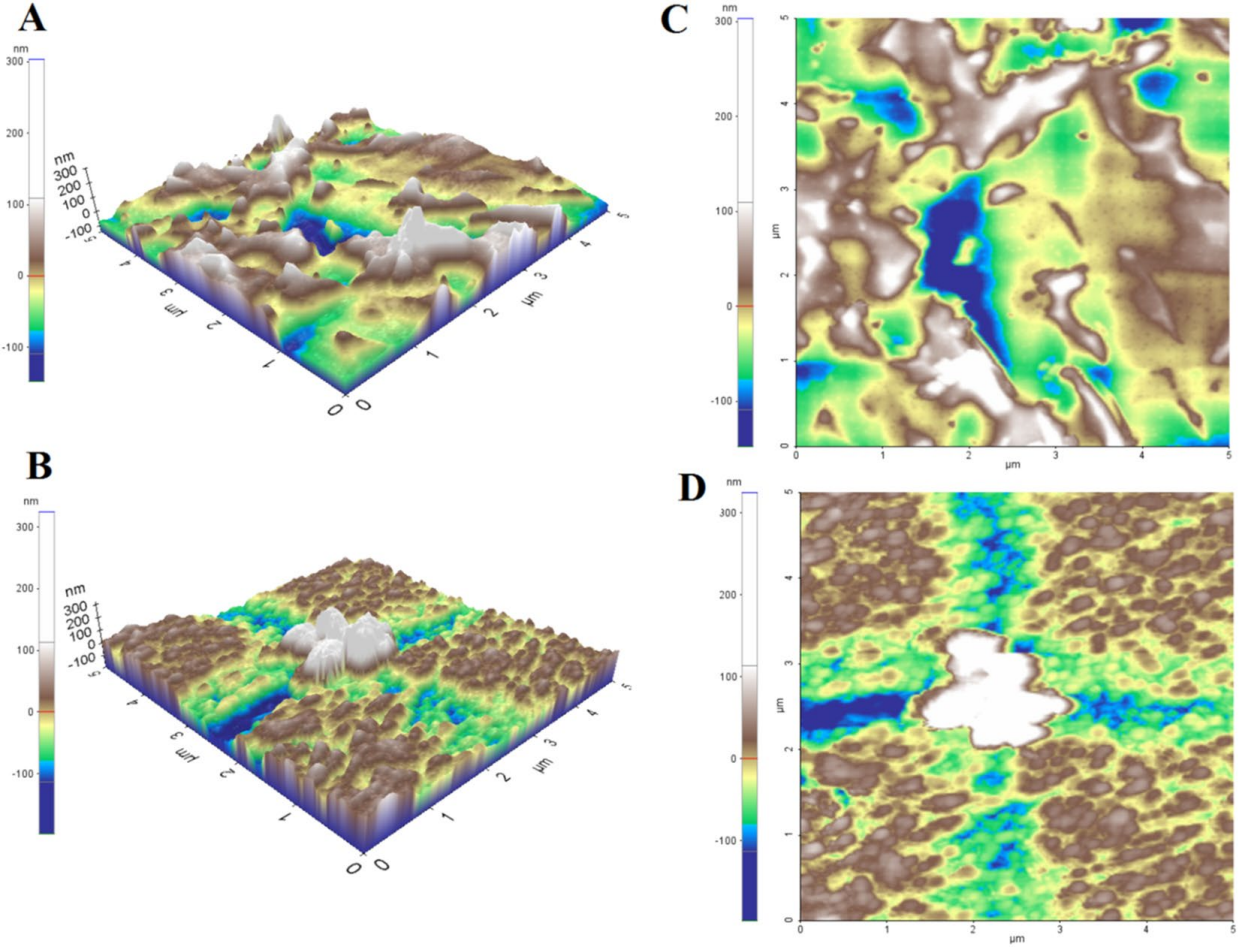
AFM images of l-EPSs [l-EPS_B3_ (**A,C**) and l-EPS_GD2_ (**B,D**); three (**A,B**) and two dimensional views (**C,D**)].

The topographical AFM images of l-EPSs are shown in Fig. 2A–D. The surface morphology of the l-EPSs obtained from *L. delbrueckii* ssp. *bulgaricus* B3 and *L. plantarum* GD2 strains is rough and porous. The maximum height of the surface morphology is 303 and 324 nm and the minimum depth is 147 and 197 nm, respectively. The average roughness of surface morphology is 77 and 63 nm, respectively. Ahmed *et al*.^49^, Piermaria *et al*.^50,51^ reported smooth and glittering surface for *L. kefiranofaciens* ZW3 EPS and kefiran grain, respectively. Wang *et al*.^44^ reported a smooth and porous surface for *L. plantarum* KF5 EPS. Cell attachment depends on the surface properties of the polymers, such as roughness, topography, and charge^52^. The rough surface morphology provides greater attachment capabilities compared to smooth surface morphology^53^. Moreover, surface roughness has also been demonstrated to have a potentially significant effect on cell adhesion^54^. *L. delbrueckii* ssp. *bulgaricus* B3 showed the best adhesive ability (88%); There was also a positive correlation between the two groups (*p* < 0.05). Following *L. delbrueckii* ssp. *bulgaricus* B3 strain, the GD2 strain showed strong adhesion (83%) in our previous study (not published data). The surface morphology of l-EPS obtained from *L. delbrueckii* ssp. *bulgaricus* B3 shows a greater average roughness than the l-EPS originated from *L. plantarum* GD2. Therefore, the attachment capability of l-EPS obtained from *L. delbrueckii* ssp. *bulgaricus* B3 may be higher. The results obtained from our previous study also support this conclusion.

**Figure 3:**
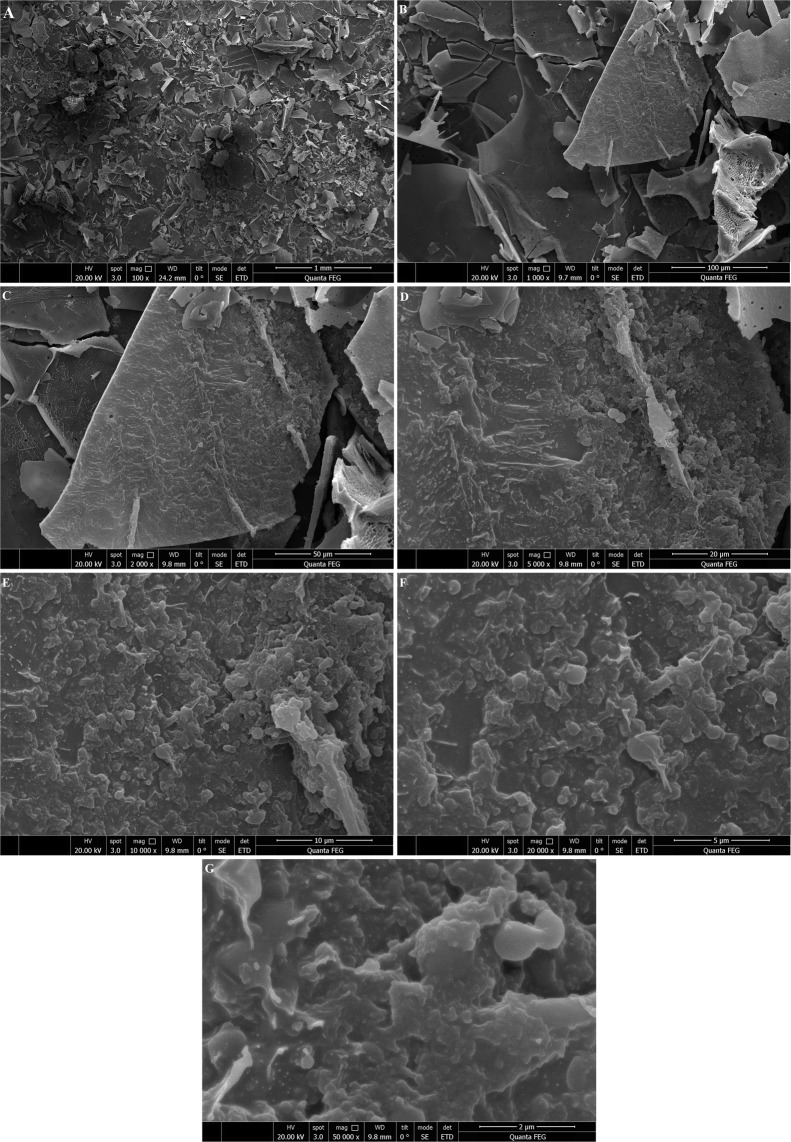
SEM images of l-EPS_B3_. Fig (**A–G**) showing SEM images of l-EPS_B3_ at 100×, 1000×, 2000×, 5000×, 10.000×, 20.000×, respectively.

**Figure 4:**
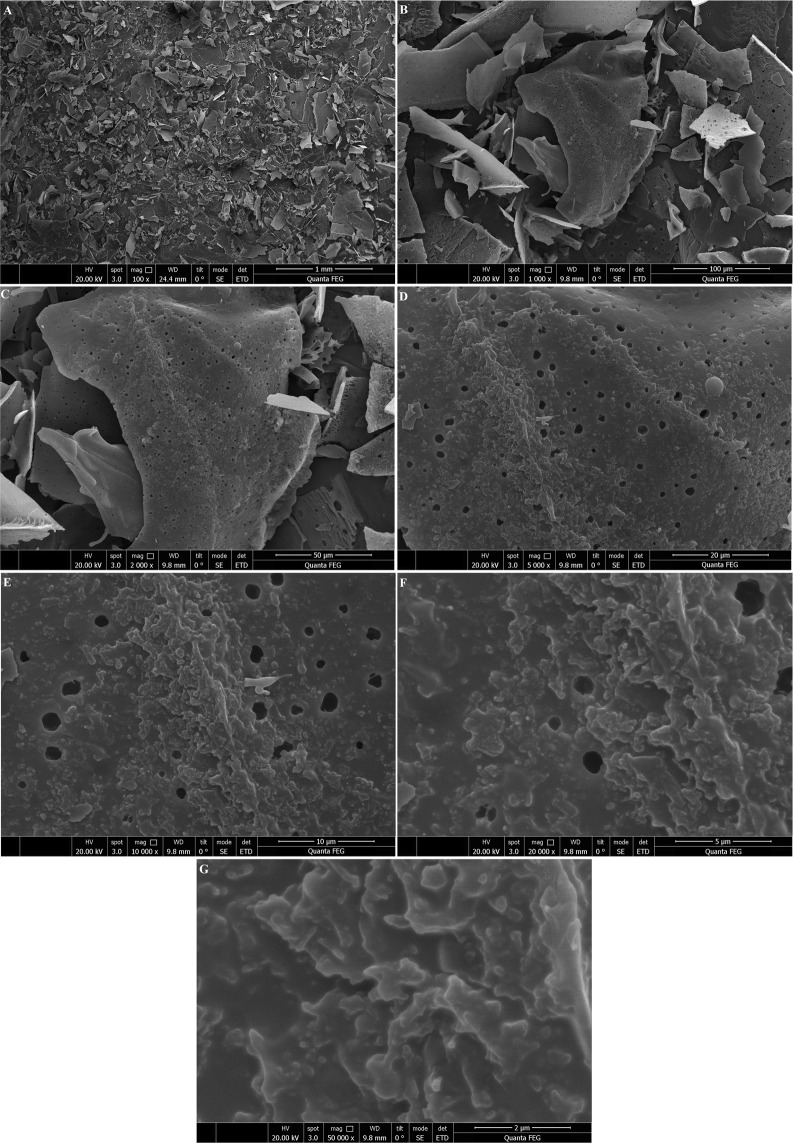
SEM images of l-EPS_GD2_. Fig (**A–G**) showing SEM images of l-EPS_GD2_ at 100×, 1000×, 2000×, 5000×, 10.000×, 20.000×, respectively.

In addition to AFM, another very commonly used tool for EPS imaging is SEM, as reported by many researchers. SEM is a very useful tool to study the surface topography of polymers^49^. SEM results of l-EPSs obtained from *L. delbrueckii* ssp. *bulgaricus* B3 and *L. plantarum* GD2 strains are shown in Figs. 3 and 4. The surface morphology of the l-EPSs obtained from *L. delbrueckii* ssp. *bulgaricus* B3 and *L. plantarum* GD2 strains was found to be rough and porous, as done in AFM.

**Figure 5:**
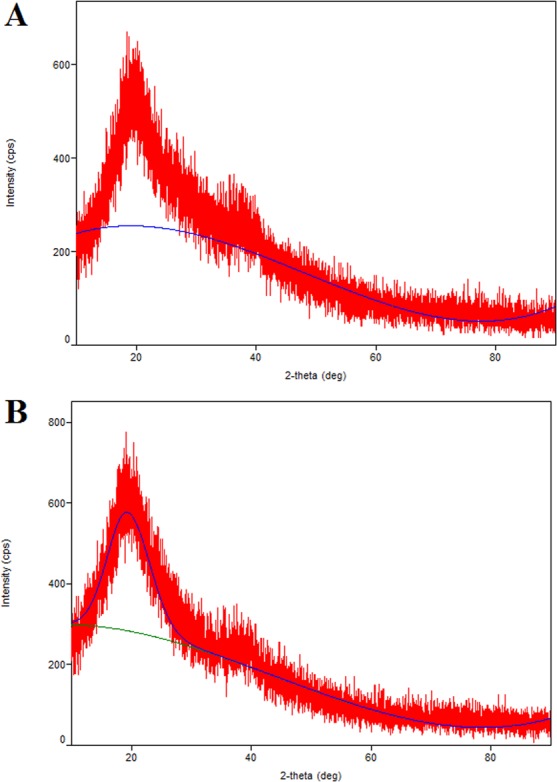
XRD pattern of l-EPSs [l-EPS_B3_ (**A**) and l-EPS_GD2_ (**B**)].

XRD is a quite potent analytical technique that is commonly used for phase identification of materials^55^. As compared to XRD patterns, l-EPSs originated from *L. delbrueckii* ssp. *bulgaricus* B3 and *L. plantarum* GD2 strains had a largely amorphous or weak micro-arrangement (weak crystalline) structure (Fig. 5A,B). A study by Du *et al*.^41^ demonstrated that EPS originated from *Schizophyllum commune* has a primarily amorphous character structure as per the XRD pattern. We found XRD patterns of l-EPSs obtained from *L. delbrueckii* ssp. *bulgaricus* B3 and *L. plantarum* GD2 strains that were consistent with those reported in the literature. As compared with the corresponding ordered crystalline counterpart, amorphous material more quickly dissolves and offers a greater solubility and bioavailability. A superior biological activity often appears as a favorable property of the amorphous form of a material^56^.

DPPH free radical is a stable radical that carries an unpaired valence electron at a single atom on the nitrogen bridge^14^. The interaction of antioxidants with a DPPH free radical result in the transfer of either an electron or a hydrogen atom from the former to the latter, resulting in the neutralization of its free radical properties^57^. Table 3 demonstrates the concentration dependent scavenging activity of l-EPSs against DPPH free radicals (*p* < 0.05). The free radical scavenging activity of l-EPS_B3_ and l-EPS_GD2_ was 58% and 53% of DPPH, respectively, at a concentration of 1250 µg/mL of both compounds. The free radical scavenging activity of ascorbic acid at a concentration of 0.5 mg/mL was 95% of DPPH. A report by Zhang *et al*.^14^ indicated that a concentration of 4 mg/mL of LPC-1 of *L. plantarum* C88 in origin had a free radical scavenging activity of 52% of DPPH. A study by Liu *et al*.^58^ demonstrated a concentration of 10 mg/mL of 101EP and 102EP originated from *L. paracasei* subsp. *paracasei* NTU 101 and *L. plantarum* NTU 102 scavenged 92% and 81% DPPH free radicals, respectively. Our study demonstrated considerably higher scavenging activity of l-EPSs for DPPH free radical compared to that shown for LPC-1, 101EP, and 102EP by previous studies.

**Table 3:** Antioxidant activity of l-EPSs.

	Antioxidant activity									
	DPPH free radical scavenging (%)		Hydroxyl radical scavenging (%)		Lipid peroxidation inhibitory (%)		Metal ion chelating (%)		Superoxide anion scavenging (%)	
**Positive control**	95 ± 4		60 ± 2		25 ± 1		94 ± 3		98 ± 2	
	**l-EPS**_**B3**_	**l-EPS**_**GD2**_	**l-EPS**_**B3**_	**l-EPS**_**GD2**_	**l-EPS**_**B3**_	**l-EPS**_**GD2**_	**l-EPS**_**B3**_	**l-EPS**_**GD2**_	**l-EPS**_**B3**_	**l-EPS**_**GD2**_
**100 µg/mL**	6 ± 1^b,c,d,e^	3 ± 1^b,c,d,e^	7 ± 1^b,c,d,e^	5 ± 1^b,c,d,e^	12 ± 0^b,c,d,e^	10 ± 0^b,c,d,e^	13 ± 2^b,c,d,e^	11 ± 0^b,c,d,e^	3 ± 0^b,c,d,e^	2 ± 1^b,c,d,e^
**250 µg/mL**	14 ± 2^a,c,d,e^	12 ± 2^a,c,d,e^	13 ± 0^a,c,d,e^	7 ± 2^a,c,d,e^	19 ± 2^a,c,d,e^	16 ± 2^a,c,d,e^	18 ± 1^a,c,d,e^	14 ± 2^a,c,d,e^	8 ± 1^a,c,d,e^	6 ± 0^a,c,d,e^
**500 µg/mL**	25 ± 3^a,b,d,e^	23 ± 1^a,b,d,e^	24 ± 2^a,b,d,e^	16 ± 0^a,b,d,e^	28 ± 1^a,b,d,e^	24 ± 2^a,b,d,e^	28 ± 1^a,b,d,e^	26 ± 1^a,b,d,e^	19 ± 2^a,b,d,e^	16 ± 3^a,b,d,e^
**1000 µg/mL**	42 ± 2^a,b,c,e^	38 ± 2^a,b,c,e^	36 ± 1^a,b,c,e^	34 ± 2^a,b,c,e^	37 ± 1^a,b,c,e^	36 ± 1^a,b,c,e^	38 ± 2^a,b,c,e^	34 ± 2^a,b,c,e^	36 ± 3^a,b,c,e^	27 ± 2^a,b,c,e^
**1250 µg/mL**	58 ± 1^a,b,c,d^	53 ± 1^a,b,c,d^	54 ± 2^a,b,c,d^	48 ± 2^a,b,c,d^	48 ± 2^a,b,c,d^	42 ± 1^a,b,c,d^	46 ± 2^a,b,c,d^	43 ± 3^a,b,c,d^	48 ± 2^a,b,c,d^	36 ± 1^a,b,c,d^

Values are expressed as mean ± SD. Tukey’s test, if p < 0.05.

^a^100 µg/mL l-EPSs (n:3 for each row), ^b^250 µg/mL l-EPSs (n:3 for each row), ^c^500 µg/mL l-EPSs (n:3 for each row), ^d^1000 µg/mL l-EPSs (n:3 for each row), ^e^1250 µg/mL l-EPSs (n:3 for each row).

Hydroxyl radical, a potent oxidant molecule, can enter reaction with nearly all biological compounds (proteins, lipids, and carbohydrates)^4^. H_2_O_2_ molecules can indirectly generate oxidative injury in biomolecules through the generation of hydroxyl radical *via* Fenton reaction and/or Haber–Weiss reaction using iron as a catalyzer; antioxidants are capable of preventing and/or inhibiting those reactions^57^. Table 3 shows that l-EPSs can scavenge hydroxyl radicals in a concentration dependent manner, an effect that rises in case of a concentration ranging from 100 to 1250 µg/mL (*p* < 0.05). l-EPS_B3_ and l-EPS_GD2_ had a hydroxyl radical scavenging activity of 54% and 48%, respectively, at a concentration of 1250 μg/mL, at a concentration of 0.5 mg/mL, ascorbic acid had a hydroxyl radical scavenging activity of 60%. According to a report by Pan and Mei^7^, a concentration of 10 mg/mL on the EPS-I originated from *Lactococcus lactis* subsp. *lactis* 12 demonstrated a hydroxyl radical scavenging activity of 75%. As Guo *et al*.^59^ reported, at a concentration of 10 mg/mL, EPS and Se-EPS of *Lactococcus lactis* subsp. *lactis* origin yielded a hydroxyl radical scavenging activity of 55% and 70%, respectively. We demonstrated higher hydroxyl scavenging activity of l-EPSs than reported by others for EPS-I, EPS, and Se-EPS. EPSs hydroxyl radical scavenging activity derives from a readily occurring anomeric hydrogen abstraction from the internal monosaccharide units^60^. Hence, the above-mentioned property may have increased hydroxyl radical scavenging activity of our l-EPSs.

It has been previously reported that the hydroxyl radical scavenging activity of natural EPSs does not directly stem from the scavenging activity rather, it is dependent on inhibitory effects of chelating ions, such as iron (II) and copper (II) on hydroxyl radical generation. Metal ion chelating activity may be involved in hydroxyl radical scavenging exerted by EPSs, an effect made evident by concentration reduction affecting the catalyzing transition metal during lipid peroxidation^7^.

Lipid peroxidation is a form of oxidative alteration of polyunsaturated fatty acids found in the cell membranes, resulting in the formation of several degradation products such as MDA that can enter a chemical reaction with TBA^61^. The iron/H_2_O_2_ system was used to catalyze the oxidation reaction while the MDA level indicated the extent of lipid peroxidation. Thus, anti lipid peroxidation activity was closely linked to hydroxyl radical scavenging activity^60^. Table 3 depicts the inhibitory activity of l-EPSs on lipid peroxidation. An inhibitor activity of lipid peroxidation was shown in a dose dependent fashion for both l-EPSs (*p* < 0.05). l-EPS_B3_ possessed a lipid peroxidation inhibitory activity of 48% and l-EPS_GD2_ 42% for a concentration of 1250 µg/mL. The corresponding activity for ascorbic acid at a concentration of 0.5 mg/mL was 25%. Li *et al*.^4^ reported that lipid peroxidation inhibitory activities of 37% and 33% for B-EPS and L-EPS, respectively, which were isolated from *L. plantarum* R315 at a concentration of 1.50 mg/mL. For a concentration of 0.1 mg/mL, EPSa and EPSb originated from *Bifidobacterium animalis* RH had an inhibitory effect of 33% and 52%, respectively, as Xu *et al*.^62^ reported. We detected higher lipid peroxidation inhibitory activity of l-EPSs than B-EPS and L-EPS used in other studies. However, our l-EPSs in our study showed a reduced lipid peroxidation inhibitory activity than that shown by others for EPSa and EPSb studies.

It has been argued that metal ion chelating activity reducing the amount of the catalyzing transition metal in lipid peroxidation is among other several antioxidant mechanisms. Ferrous ion, one of the available transition metals, is well known for its high reactivity potential and thus considered as the most notable lipid oxidation prooxidant. Metal ion chelating agents create s bonds with metal and are efficient secondary antioxidants since they can reduce the redox potential and thus stabilize the oxidized form of the iron ion^57^. Table 3 shows that iron (II) chelation activity of 1250 µg/mL l-EPS_B3_ and l-EPS_GD2_ were 46% and 43%, respectively. EDTA-Na, at a concentration of 0.5 mg/mL, had an iron-chelating potency of 94%. A study by Li *et al*.^57^ demonstrated up to 59%, 53%, 46% and 39% metal ion chelating activity at a concentration of 4 mg/mL for crude EPS, EPS-1, EPS-2 and EPS-3 isolated from *L. helveticus* MB2-1, respectively. 101EP and 102EP isolated from *Maxwell Bay, King George Island, indicating the paracasei* subsp. *paracasei* NTU 101 and *L. plantarum* NTU 102, in a study by Liu *et al*.^58^, demonstrated 54% and 29% metal ion chelating activity, respectively, at a concentration of 10 mg/mL. l-EPSs employed by our study had higher metal ion chelating activity than what has been reported for EPS, 101EP, and 102EP.

The superoxide radical was formed in a phenazine methosulphate (PMS)/NADH reduced system and assayed by the reduction of NBT^63^. Table 3 shows that the l-EPSs scavenged superoxide radical depending on their concentration (*p* < 0.05). A concentration of 1250 µg/mL for both l-EPS_B3_ and l-EPS_GD2_ enabled both l-EPSs to show superoxide radical scavenging activity reaching 48% and 36%, respectively. Ascorbic acids superoxide anion scavenging activity reached 98% for a concentration of 0.5 mg/mL. In a study by Wang *et al*.^8^, EPS isolated from *L. plantarum* YW32 had a superoxide anion scavenging activity of 67% at a concentration of 5 mg/mL. EPS115 and EPS111 isolated from *L. rhamnosus* used in a study by Rajoka *et al*.^64^ had a 6% and 29% superoxide anion scavenging activity, respectively, at a concentration of 4 mg/mL. Our l-EPSs showed a greater percent superoxide anion scavenging activity than EPS, EPS115 and EPS111 used in other studies. Superoxide anion scavenging activity may occur through the dissociation energy of the OH bond. As the number of electron-withdrawing groups bound to polysaccharide increases, the dissociation energy of the OH bond is reduced. Some sort of the electrophilic groups such as aldehyde or keto that facilitate dissociation of hydrogen from OH bond has been linked to an effective superoxide anion scavenging^57^. Hence, l-EPSs showed promising antioxidant activity (DPPH free radical scavenging activity, hydroxyl radical scavenging activity, metal ion chelating activity, lipid peroxidation inhibitory activity, and superoxide anion scavenging activity).

Aβ_1-42_ (10-100 μM) was used on SH-SY5Y cells for 24 h after which MTT assay determined survival of cells. Ten to 100 μM of Aβ_1-42_ caused significant reduction of the survival of cells based on the dose used (*p* < 0.05). Aβ_1-42_, at a dose of 10 μM caused the death of 45% of the cells in comparison to control cells (data not demonstrated). Therefore, a 24 h treatment of 10 μM Aβ_1-42_ was used for creating SH-SY5Y cell injury in the next experiments. Wei *et al*.^65^ as well as Wang *et al*.^66^ demonstrated that Aβ_1-42_ exerted cytotoxicity on SH-SY5Y cells.

The administration of Aβ_1-42_ (10 μM for 24 h) caused a substantial drop in cell viability to 55% (*p* < 0.05), whereas l-EPSs significantly prevented Aβ_1-42_-mediated cell damage (*p* < 0.05), restoring cell survival to a range between 59% and 85% (data not shown). This is the first study that reports that l-EPSs obtained from *L. delbrueckii* ssp. *bulgaricus* B3 and *L. plantarum* GD2 act to protect cells from Aβ-mediated cellular toxicity. Recent research has demonstrated the protective effect of *L. plantarum versus pentosus* in memory impairment in mice with D-Gal- and scopolamine-induced AD^11,12^. The protective effect of l-EPSs obtained from *L. delbrueckii* ssp. *bulgaricus* B3, and *L. plantarum* GD2 strains on SH-SY5Y cells was in line with the literature^11,12^.

l-EPSs at different concentrations alone did not cause any apparent cytotoxicity (data not shown). Ismail and Nampoothiri^67^ reported that 50 mg/mL EPS obtained from *L. plantarum* MTCC 9510 strain had a cytotoxic effect on normal fibroblast cells (L929) at 60%. Dilna *et al*.^68^, according to the study, the EPS obtained from *L. plantarum* RJF4 strain at a concentration of 500 µg/mL normal fibroblast cells (L6) on the 35% cytotoxic effect was determined. The cytotoxic effect of l-EPSs obtained from *L. delbrueckii* ssp. *bulgaricus* B3, and *L. plantarum* GD2 strains on SH-SY5Y cells was in line with the literature^67,68^.

Aβ is traditionally considered a major factor in the development of AD. Aβ deposition found in senile plaques has a significant association with reactive oxygen species (ROS), which are thought to play a major role in degenerative events. Oxidative stress leads to cellular accumulation of oxidized biomolecules and ROS, which in turn stimulates the antioxidant defense system. Meanwhile, nuclear factor erythroid 2-related factor 2 (Nrf2) causes some antioxidant enzyme to be activated, leading to the breakdown of accumulated ROS. Increased mitochondrial ROS production boosts the activity of recombinant human beta-site amyloid precursor protein (APP)-cleaving enzyme 1 (BACE1), resulting in the augmentation of amyloidogenic APP processing and thus Aβ overproduction^69^.

**Figure 6:**
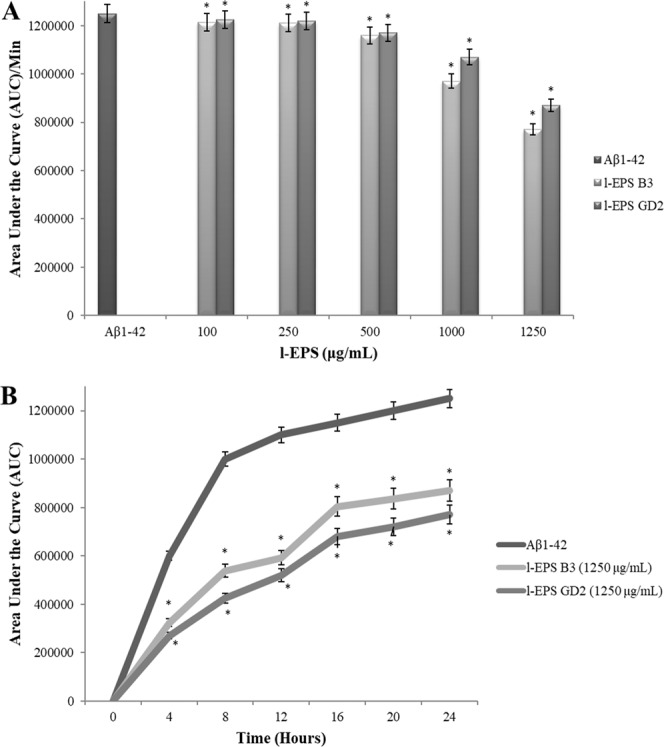
Dose-dependent inhibition of Aβ_1-42_ aggregation by l-EPSs (**A**), time course of inhibition of Aβ_1-42_ aggregation by l-EPSs (**B**) using the ThT assay. Values are expressed as mean ± SD. Pearson’s correlation test, if *p* < 0.05. *significant difference from the Aβ_1-42_ (n:3 for each bar).

**Figure 7:**
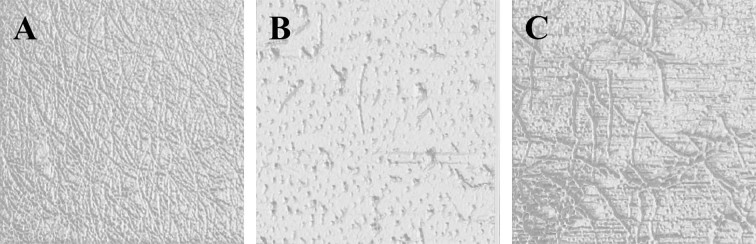
AFM images of Aβ_1-42_ fibrils formed when Aβ_1-42_ was incubated alone (**A**) or in the presence of l-EPS_B3_ (**B**) and l-EPS_GD2_ (**C**) at 37 °C for 24 h. The figure size was 10 µm × 10 µm.

A anti-aggregative effect of l-EPSs against Aβ_1-42_, if any, was investigated using the ThT assay. Fibrils formation by Aβ_1-42_ was demonstrated by the ThT assay through a significant 24 h increase in fluorescence (*p* < 0.05); Aβ_1-42_ fibril formation was inhibited by l-EPSs proportionally to their concentration during the same period (*p* < 0.05). The most strong anti-aggregant effect was shown by l-EPS_B3_ at 1250 µg/mL, which was evidenced by an almost 40% intensity reduction in ThT fluorescence (Fig. 6A,B). Compatible with the results of the ThT assay, the findings of AFM suggested that l-EPSs can directly prevent the fibrils from the formation of Aβ_1-42_. We demonstrated a direct and dose dependent inhibitory action of l-EPSs on Aβ_1-42_ aggregation (Fig. 7A–C). As far as we know, the overall results of the present study indicates for the first time in the literature that l-EPSs exert an anti-aggregant effect against Aβ_1-42_. Olasehinde *et al*.^70^ indicated that ThT assay showed a high fluorescence intensity upon incubation of Aβ_1-42_ peptide without the polysaccharides although treatment of Aβ_1-42_ with *Ecklonia maxima* (PMNP), *Gelidium pristoides* (PKPM), and *Ulva rigida* (PURL) produced a significant decrease in fluorescence intensity. Persistent aggregation of Aβ fibrils was also shown in the control (Aβ_1-42_ alone) by SEM. Nevertheless, incubation of sulfated polysaccharides samples with Aβ_1-42_ eliminated Aβ_1-42_ fibrils, suggesting that the fibrils were broken down and their aggregation was inhibited. Zhou *et al*.^71^ demonstrated a significant benefit of neuropathology with the addition of polysaccharides from *Lycium barbarum* (LBP1), the latter also improved cognitive functions and restored synaptic plasticity in APP/PS1 transgenic mice by eliminating amyloid pathology and favoring neurogenesis. LBP1 reportedly reduces Aβ peptide production and aggregation *in vitro*. According to a report by Li *et al*.^69^, *Amanita caesarea* polysaccharides (ACPS) improve the antioxidant defense systems through increasing superoxide dismutase (SOD) and glutathione peroxidase (GSH-Px) levels; a significant reversal of the enhanced ROS level occurred in the mice with AD. They stated that altertation of Nrf2-mediated oxidative stress by ACPS at least partially explains their protective effects against AD. ACPS protected against apoptosis in an L-Glutamine (L-Glu)-induced cell apoptosis model and in mice with AD induced by D-Gal and aluminum chloride (AlCl_3_). In our opinion, there may occurs a direct inhibition of the progression of fibril formation by l-EPSs, which also destabilize Aβ_1-42_ fibrils or inhibit their aggregation, or a alternatively, modulate the antioxidant defense system to reduce Aβ production in an indirect manner.

**Figure 8:**
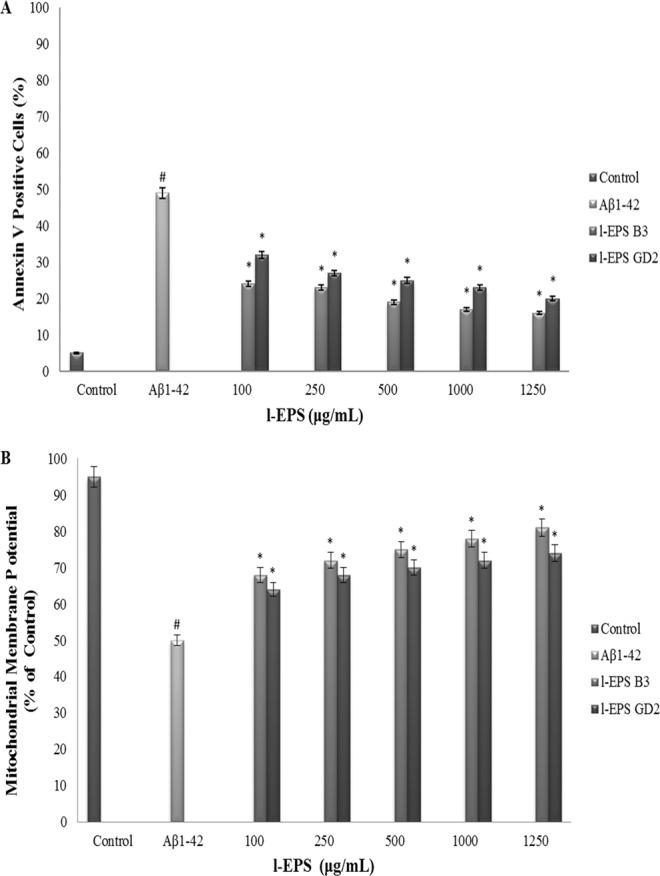
Effect of l-EPSs on Aβ_1-42_-induced cell apoptosis (**A**) and the loss of Δψm and the release of mitochondrial cytochrome *c* into the cytosol (**B**) in SH-SY5Y cells using flow cytometry. Values are expressed as mean ± SD. Pearson’s correlation test, if *p* < 0.05. #significant difference from the control (n:3 for each bar). *significant difference from the Aβ_1-42_ (n:3 for each bar).

In order to corroborate the above finding, the flow cytometric analysis was done to rate the l-EPSs potential to protect SH-SY5Y cells against Aβ_1-42_-mediated apoptosis. Figure 8A shows that freshly cultured SH-SY5Y contained a percentage of living SH-SY5Y cells (95%) and a relatively lower proportion of dead cells (5%). Unlike that finding, cell death was significantly different (*p* < 0.05) after being treated with 10 μM Aβ_1-42_ for 24 h, where the lowest mean proportion of living SH-SY5Y was 51% while the highest proportion of apoptotic SH-SY5Y was 49%. Aβ_1-42_ and l-EPSs led to significantly lower apoptotic fractions of SH-SY5Y compared to the exposure to Aβ_1-42_ (*p* < 0.05). In particular, cell death rate dropped from 49% to 16%. These data suggest that l-EPSs were quite potent at eliminating cytotoxic effect of Aβ_1-42_ while protecting SH-SY5Y cells leading to a decrease of cell death rate by 33%. The anti-apoptotic effects of EPSs have been shown by various studies in the literature. Zhang *et al*.^72^ demonstrated a significant decrease in the rate of hepatic cell death in rats with obstructive jaundice after treatment with oral *L. plantarum*. Lam *et al*.^73^ reported a significant reduction of ethanol-induced mucosal lesion area and gastric cell apoptosis among rats pre-treated with *L. rhamnosus* GG. Sunanliganon *et al*.^74^ revealed that *L. plantarum* B7 could show an anti-*Helicobacter pylori* effect *in vitro* and, improve gastric histopathology by exerting various anti-inflammatory actions on *H. pylori* infection, as well as reducing serum TNFα levels, gastric malondialdehyde (MDA), and epithelial cell apoptosis. The study of Bouhafs *et al*.^75^ showed that the administration of *L. plantarum* BJ0021 decreased cell death, suggesting a potential protective role in reducing the toxicity of endosulfan (EDS) in pregnant rats. The anti-apoptotic effect of l-EPSs obtained from *L. delbrueckii* ssp. *bulgaricus* B3, and *L. plantarum* GD2 strains on SH-SY5Y cells was in line with the literature^73–75^.

**Figure 9:**
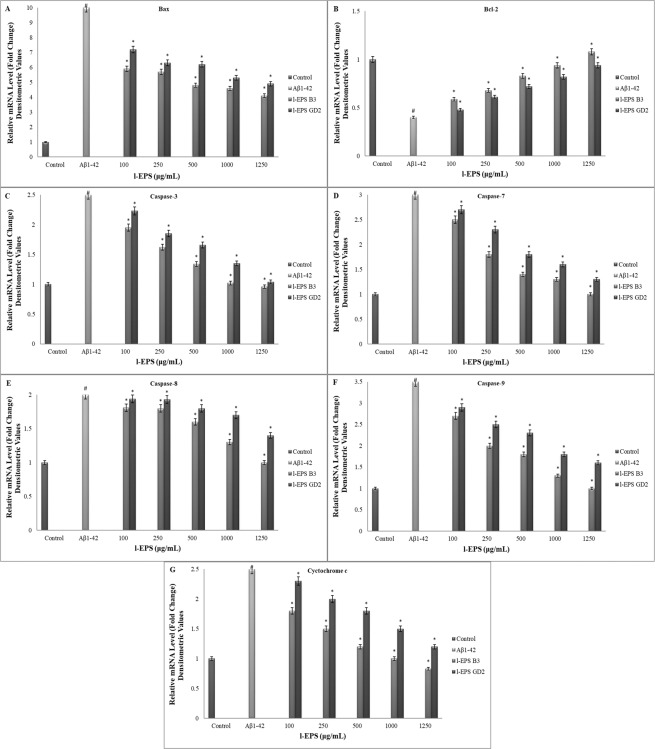
The mRNA levels of Bax (**A**), Bcl-2 (**B**), caspase-3 (**C**), caspase-7 (**D**), caspase-8 (**E**), caspase-9 (**F**), and cyctochrome *c* (G) in SH-SY5Y cells. Values are expressed as mean ± SD. Pearson’s correlation test, if *p* < 0.05. #significant difference from the control (n:3 for each bar). *significant difference from the Aβ_1-42_ (n:3 for each bar).

Loss of Δψm occurs early in the process of apoptosis induced by a number of stimuli. Δψm was detected with JC-1, the stain utilized to rate mitochondrial membrane depolarization, in order to study if the Δψm pathway is involved in Aβ_1-42_-induced cell death or is changed by l-EPSs in the presence of Aβ_1-42_. As shown in Fig. 8B, cells treated with Aβ_1-42_ showed a significant increase in mitochondrial depolarization; the percentage of cells that result positive for depolarized mitochondria increased from 5% of the control cells to 50% of the cells treated with 10 μM Aβ_1-42_ (*p* < 0.05). Mitochondrial depolarization leads to the release of several apoptogenic proteins, most notably cytochrome *c*, from the mitochondrion into the cytosol. RT-PCR revealed that 10 μM Aβ_1-42_ led to an accumulation of cytochrome *c* in the cytosol for 24 h **(**Fig. 9G**)**. The amount of cytochrome *c* observed in the cytosol was significantly reduced when the cells were treated with l-EPSs before Aβ_1-42_ treatment (*p* < 0.05). Pretreatment with l-EPSs protected the cells against Aβ_1-42_-induced decrease of mitochondrial membrane potential, reducing the number of JC-1 negative cells. These results indicate that Aβ_1-42_ causes a mitochondrial dysfunction via the mitochondrial pathway, and l-EPSs have a protective effect against such dysfunction. The protective effect against such mitochondrial dysfunction of EPSs have been shown by various studies in the literature. Peng *et al*.^13^ reported that *L. plantarum* NDC75017 was able to relieve learning and memory-associated injuries in aging rats by reducing D-Gal-induced mitochondrial dysfunction. A study by Li *et al*.^76^ provided direct evidence of a concentration-dependent ability of *Cordyceps militaris* polysaccharides (CMP) to inhibit Fe^2+^-L-Cysteine-induced mitochondrial injury and swelling, as well as a marked potential for scavenging superoxide anions. Furthermore, CMP significantly increased the activities of SOD, catalase (CAT), GSH-Px, and anti-hydroxyl radicals in liver mice. This was suggestive of a protective effect of CMP on mitochondria, by virtue of scavenging ROS, preventing mitochondrial swelling, and augmenting the activities of antioxidases. Hence, CMP may show pharmaceutical promise for use in mitochondrial protection and anti-aging. The protective effect against such mitochondrial dysfunction of l-EPSs obtained from *L. delbrueckii* ssp. *bulgaricus* B3, and *L. plantarum* GD2 strains on SH-SY5Y cells was in line with the literature^13,76^.

The intrinsic pathway of the cell death is closely associated with the Bcl families anti-apoptotic activity. It has been reported that the translocation of the pro-apoptotic Bax gene to the mitochondrial membrane with resulting depolarization and cytochrome *c* release activates cell death^77^. A number of signal cascades regulate cell death at specific conditions. The caspase cascade system plays a crucial role for the induction, transduction, and amplification of signals leading to cell death within a given cell^78^.

In order to better delineate the protective action of l-EPSs with regard to Aβ_1-42_-mediated cell death, seven genes linked with Aβ_1-42_-mediated cell death were studied in depth. Figure 9 shows that when 10 μM Aβ_1-42_ is added, it reduced Bcl-2 mRNA expression (Fig. 9A), augmented mRNA expression of Bax (Fig. 9B), caspase-3 (Fig. 9C), caspase-7 (Fig. 9D), caspase-8 (Fig. 9E), caspase-9 (Fig. 9F), and cytochrome *c* **(**Fig. 9G**)**. In line with the expectations, SH-SY5Y cells exposure to Aβ_1-42_ with l-EPSs found in the medium significantly augmented mRNA expression of Bcl-2 than those observed solely with Aβ_1-42_ treatment (*p* < 0.05); however, it significantly reduced Bax, caspase-3, caspase-7, caspase-8, caspase-9, and cytochrome *c* activations (*p* < 0.05).

EPSs reportedly scavenge free radicals *in vitro* and mitigate oxidative injury in various cells and animal models while they exert *in vivo* antioxidant capacity by regulating peroxidation products, antioxidant defense system, and stress-related signaling (like apoptosis). Since oxidative stress is strongly related to neurodegeneration, several antioxidant EPSs have been studied for anti-AD activity in multiple neurodegenerative models, which revealed that they mitigate neuronal injury and dysfunction. EPSs exert protective effects in association with elimination of multiple stress sources such as oxidative, inflammatory, and proteotoxic stresses^47^. Hence, the evidence was provided about the important protective role of l-EPSs protecting from Aβ_1-42_-mediated cell death. This protective action of l-EPSs sparing SH-SY5Y cells from Aβ-induced cytotoxicity is linked with the biological activity of l-EPSs. l-EPS obtained from *L. delbrueckii* ssp. *bulgaricus* B3 proven to be more effective against Aβ_1-42_-mediated cell death as well as mitochondrial depolarization. l-EPSs obtained from B3 strain can be effective due to the high mannose ratio, high molecular weight, presence of polyanionic functional groups, rough surface morphology, and amorphous character structure.

## Conclusion

In conclusion, our results showed that l-EPSs have a protective effect caused by Aβ_1-42_ neurotoxicity in SH-SY5Y cells. l-EPSs reduced neuronal cell death and inhibition of stabilizing mitochondrial function. Mannose ratio, molecular weight, functional groups, surface morphology, and amorphous character structure of l-EPSs are thought to play a role in the protective effect of l-EPSs. These observations contribute the inclusion of l-EPSs among the therapeutic options used to manage various neurological disorders in the traditional medicine in a scientific manner, indicating that l-EPSs may be promising natural chemical constituents that warrant advanced research and development for pharmacological therapy of AD.

## Data Availability

All data generated or analysed during this study are included in this published article (and its Supplementary Information files).
